# A Complete Treatise on Midwifery; or the Theory and Practice of Tokology; Including the Diseases of Pregnancy, Labor, and the Puerperal State

**Published:** 1852-01

**Authors:** 


*A Complete Treatise on Midwifery: or the Theory and Practice of Tokology: including the diseases of Pregnancy, Labor, and the Puerperal state.* By ALF. A. L. M. VELPEAU, M. D. Translated from the French by CHARLES D. MEIGS, M. D., Professor of Midwifery in the Jefferson Medical College, &c. *Fourth American, with the additions from the last French edition,* by WM. BYRD PAGE, M. D., Lecturer on Obstetrics in the Philadelphia Medical Institute, &c. With numerous illustrations. Philadelphia: Lindsay & Blakiston, 1852.

We are pleased to welcome with the opening year a new edition of this most excellent and "*complete*" treatise on Midwifery. It justly takes rank amongst the most popular of its kindred, as is evidenced by the demand for a *fourth* American, from the *second* French edition. The preparation of the present issue has been confided to Dr. Page, a gentleman in every way competent to the task, who has introduced all the modifications of the author in the last edition, at the same time retaining the notes of Dr. Meigs. The work has been so thoroughly reviewed in previous numbers of this Journal, that a brief enumeration of the changes is all that our present limits will permit. The author details his emendations as follows:

The first edition of this book was only the recapitulation of my lectures. I had particularly designed it for the use of students. I have in this endeavored to present, as nearly as possible, the actual state of the science, under the double aspect of theory and practice—my aim being to render it useful, not only to students, but also to practitioners, and even to those who are engaged in teaching this science.

The article concerning the pelvis is here entirely new in relation to its axis, its straits especially, and its vicious conformations. It is the same with that on the genital organs, regard being had to their varieties, to the practical inductions which flow from them, and to the operations which they require.

I have completed the article on menstruation, and that on reproduction, by numerous additions; and what relates to the softening (*ramollissement*) of the symphysis during pregnancy, has been entirely rewritten.

I have added a great deal to the article on *touching*, and have endeavored to show the advantages of *touching* by the rectum, as well as of abdominal exploration, in a vast number of cases.

A long chapter upon auscultation seemed to me indispensable, and I have given it.

Extra-uterine pregnancies, their mechanism, their signs, their terminations, and especially their treatment, form the subject of an article almost entirely new.

False pregnancies, as well as double pregnancies, and superfœtation in general, are treated in the same manner. I have also renewed the article on abortion, especially in regard to the diseases of the ovum, of moles, and of the treatment required.

After these, the principal changes in this edition are :—

1st. Upon the mechanism of labor in general.

2d. Upon the mechanism of labor by the head—by the vertex first—then by the face, and finally by the pelvis: all subjects which have been discussed for fifteen or twenty years in foreign countries, and of which I intend to give a review, together with their explanation.

3d. Upon the assistance which the woman may require in natural labor, and particularly upon the use of ergot.

4th. Upon the hemorrhages (*pertes*) which occur during pregnancy, or in labor; especially upon the treatment of these affections, which I had not sufficiently dwelt upon, and of which the field has latterly been very much enlarged.

5th. Upon the shortness, too great length, twisting of the cord, and the accidents which may result from them.

6th. Upon the different ruptures sometimes observed in the course of labor.

7th. Upon tumors of the pelvis, and calculi of the bladder.

8th. Upon the coarctations of the vulva, vagina, and mouth of the uterus.

9th. Upon prolapsus of the uterus in its gravid state, deviations of its orifice, and its obliquities, properly so called.

10th. Upon the diseases, tumors, and deformities of the child, which may become causes of difficult labor.

11th. Upon the theory of spontaneous evolution, which I had scarcely mentioned, and which I have now treated at length, insisting upon the new explanation which this strange phenomenon admits of.

12th. Upon version, either by the head, or feet, and upon the relative value of these two operations, regarded in a new light.

13th. Upon presentations of the arm, and the management which they require.

14th. Upon diet, and the production of abortion and premature labor in cases of narrowness of the pelvis; questions scarcely mentioned in the first edition, and of which I thought it necessary to treat more fully in this.

15th. Upon the Cæsarian operation, either abdominal or vaginal, and upon symphysiotomy.

16th. Upon the operation for breaking up the head of the child, on which I had only said a word, and to which I have now endeavored to give its true value.

17th. Upon the cautions required in the delivery of the afterbirth.

18th. Upon the hourglass contraction on the placenta, and its retention in the uterus.

19th. Upon the possible absorption of the afterbirth; an article entirely new.

20th. Upon hemorrhage after accouchement, and the use of injections of the umbilical cord, compression over the hypogastric region, compression of the aorta, and transfusion as means of delivering women from the dangerous consequences of this accident.

21st. Upon the attentions to be given to the child, and upon the sanguine tumors on its head, which are sometimes produced in the birth; upon the flattening of the head, the falling off of the funis, and hemorrhage at the umbilicus; upon lactation, both natural and artificial; upon the dressing of the cord, and the operation of the fillet; and upon some slight indispositions of the newly-born infant.

22d. Upon the inversion of the uterus after delivery, and the principal changes which its neck undergoes after accouchement.

23d. Upon sanguine tumors of the vulva, hemorrhoids, retention of urine, inflammation of the genital organs, and the other consequences of childbirth.

24th. Upon ruptures of the uterus and vagina; upon recto-vaginal, vesico-vaginal, and urethro-vaginal fistulas.

25th. Upon perforations of the perineum, the consequences and frequency of them.

26th. Upon the lacerations of the perineum, and their treatment.

27th. Upon the engorgement of the breast, and fissures of the nipple, and also upon some of the qualities necessary for a good wet-nurse.

The seven last articles were not treated of in the edition of 1829.

Moreover, it may readily be conceived that I have thought proper to modify, more or less, all the other articles in my book, and that I have only particularized cursorily in this place those which have been re-written or superadded.

The only point on which the work is not thoroughly posted up, is on the subject of Generation, a matter the less to be regretted, as we conceive its proper position to be in a Physiological treatise, rather than in work like that under notice.

